# Correction: Migration, acculturation, and the maintenance of between-group cultural variation

**DOI:** 10.1371/journal.pone.0216316

**Published:** 2019-04-25

**Authors:** Alex Mesoudi

S1 Fig, “Boxplot of acculturation rates,” is incorrect. Please see the complete, correct S1 Fig here.

## Supporting information

S1 Fig Boxplot of acculturation rates. (PDF)
