# Supplementary material for: Migration, acculturation, and the maintenance of between-group cultural variation
Source: PLoS One. 2018 Oct 16;13(10):e0205573. doi: 10.1371/journal.pone.0205573 (PMC6191118; doi:10.1371/journal.pone.0205573)
Supplement: S1 Table — (PDF) [file pone.0205573.s001.pdf]

S1 Table: Empirical patterns of acculturation. For most measures, migrants are intermediate between heritage and host values (where available), and/or 2nd generation migrants are closer to host values than 1st generation migrants. Either of these indicate acculturation.

Trait	Host country	2nd generation	1st generation	Heritage country	Ref
Collectivism	UK: 5.45 (sd=0.77, n=99)	5.93 (sd=0.66, n=79)	6.29 (sd=0.57, n=108)	Bangladesh: NA	[1]
Dispositional attribution	UK: 5.27 (sd=0.88, n=99)	5.14 (sd=0.79, n=79)	4.94 (sd=1.01, n=108)	Bangladesh: NA	[1]
Situational attribution	UK: 4.38 (sd=1.01, n=99)	4.87 (sd=1.04, n=79)	5.16 (sd=1.08, n=108)	Bangladesh: NA	[1]
Social closeness	UK: 4.18 (sd=1.79, n=99)	4.84 (sd=1.44, n=79)	4.93 (sd=1.87, n=108)	Bangladesh: NA	[1]
Religiosity	UK: 1.86 (sd=1.21, n=99)	4.10 (sd=1.44, n=79)	4.83 (sd=1.34, n=108)	Bangladesh: NA	[1]
Family contact	UK: 3.09 (sd=2.44, n=99)	7.11 (sd=4.83, n=79)	7.33 (sd=5.23, n=108)	Bangladesh: NA	[1]
Self-serving bias	Canada: 0.36 (sd=0.65, n=98)	0.22 (sd=0.79, n=111)	NA	Japan: -0.67 (sd=1.05, n=222)	[2]
Self-serving bias	USA: 0.94 (n=35)	0.69 (n=28)	NA	Japan: 0.56 (n=23)	[3]
Friend-serving bias	USA: 0.84 (n=35)	0.68 (n=28)	NA	Japan: 1.01 (n=23)	[3]
Self-esteem	UK: 16.33 (sd=4.19, n=381)	16.87 (sd=3.99, n=562)	NA	Hong Kong: 13.57 (sd=4.45, n=360)	[4]
Self-esteem (self-report)	USA: 5.36 (sd=0.93, n=166)	4.86 (sd=1.07, n=195)	NA	China: 4.72 (sd=0.96, n=153)	[5]
Self-esteem (spontaneous)	Canada: 3.73 (sd=4.44, n=110)	2.99 (sd=3.71, n=100)	NA	China: 1.71 (sd=2.02, n=95)	[5]
Dialectic self-perception	USA: 3.61 (sd=0.83, n=115)	3.89 (sd=0.65, n=129)	NA	China: 3.98 (sd=0.69, n=153)	[5]
Actual-ideal self discrepancy	Canada: 1.20 (sd=0.49, n=90)	1.25 (sd=0.50, n=151)	NA	Japan: 1.49 (sd=0.57, n=161)	[6]
Self-serving bias	Canada: 0.27 (sd=0.66, n=90)	-0.03 (sd=0.68, n=151)	NA	Japan: -0.18 (sd=0.76, n=161)	[6]
Life satisfaction	USA: 5.12 (sd=1.18, n=170)	4.21 (sd=1.34, n=149)	NA	China: 3.38 (sd=1.17, n=141)	[7]
Self-esteem	USA: 5.77 (sd=0.94, n=170)	5.09 (sd=1.18, n=149)	NA	China: 4.93 (sd=1.13, n=141)	[7]
Relationship quality	USA: 4.01 (sd=0.70, n=170)	3.70 (sd=0.72, n=149)	NA	China: 3.31 (sd=0.86, n=141)	[7]
Positive affect	USA: 3.60 (sd=0.58, n=170)	3.34 (sd=0.57, n=149)	NA	China: 3.07 (sd=0.60, n=141)	[7]
Negative affect	USA: 2.10 (sd=0.60, n=170)	2.36 (sd=0.71, n=149)	NA	China: 2.54 (sd=0.66, n=141)	[7]
Emotional expressiveness	USA: 4.89 (sd=0.91, n=170)	4.46 (sd=0.88, n=149)	NA	China: 3.95 (sd=0.97, n=141)	[7]
Emotional differentiation	USA: 4.56 (sd=1.06, n=170)	4.14 (sd=0.99, n=149)	NA	China: 3.78 (sd=1.32, n=141)	[7]
Optimism	USA: 4.06 (n=257)	3.08 (n=44)	NA	China: 2.46 (n=312)	[8]
Trust	Denmark: 6.80 (n=2616)	6.10 (n=35)	5.86 (n=73)	Outside Western Europe: NA	[9]
Trust	Norway: 6.61 (n=3088)	6.31 (n=17)	6.07 (n=73)	Outside Western Europe: NA	[9]
Trust	Sweden: 6.29 (n=3119)	6.01 (n=63)	5.43 (n=204)	Outside Western Europe: NA	[9]
Trust	Switzerland: 5.99 (n=2686)	5.73 (n=82)	5.54 (n=258)	Outside Western Europe: NA	[9]
Trust	Netherlands: 5.83 (n=3167)	5.58 (n=126)	5.45 (n=244)	Outside Western Europe: NA	[9]

Trust	Austria: 5.48 (n=3783)	5.10 (n=221)	4.98 (n=192)	Outside Western Europe: NA	[9]
Trust	Belgium: 5.11 (n=2909)	4.46 (n=99)	4.71 (n=125)	Outside Western Europe: NA	[9]
Trust	France: 4.94 (n=3026)	4.75 (n=194)	4.72 (n=189)	Outside Western Europe: NA	[9]
Trust	Greece: 3.49 (n=1975)	3.69 (n=169)	4.27 (n=175)	Outside Western Europe: NA	[9]

Notes For all rows, 1st generation were born in the heritage country and moved to the host country after the age of 14. 2nd generation were born in the host country to 1st generation parents. Measures are only shown if there was a statistically significant difference between either host and heritage or host and 1st generation values (otherwise there would be no scope for acculturation in the 2nd generation). Where studies report 'Asian Americans' without specifying generation, they are assumed to be 2nd generation, but could include some 1st generation. Note that values are on different scales, and are intended to provide comparisons within each row between groups, not comparisons across studies and measures. In [1], collectivism, attribution, social closeness and religiosity range from 1-7. Family contact is number of family members one sees in person in an average week. Note that Table 4 in [1] contains an error, and Non-migrants and 1st-gen closeness values are the wrong way around (Fig 2B shows the correct values). In [2], self-serving bias is the "difference between the extent to which participants viewed…positively valenced traits to be characteristic of (a) themselves and (b) their same-sex peers", where the individual ratings ranged from 1-8. In [3], self and other-enhancement were calculated from Fig 1 as the difference between mean evalation of self and other students, and best friend and other students, respectively. In [4] the self-esteem scale ranges from 0-23. In [6], actual-ideal self discrepancy is the difference between one's actualised and idealised self characteristics, and self-serving bias is the difference between one's actualised characteristics and an average other. In [5], self-esteem (self report) and dialectic self-perception range from 1-7, and self-esteem (spontaneous) is the ratio of positive to negative words in the Twenty Statements Test. In [7], measures are on scales of 1-7. In [9], Trust values are from the European Social Survey 2004-2007 and on a scale of 0-10. Only those countries are listed where [9] found a significant difference between 1st gen and host country respondents.

References

1. Mesoudi A, Magid K, Hussain D. How do people become W.E.I.R.D.? Migration reveals the cultural transmission mechanisms underlying variation in psychological processes. PLOS ONE. 2016;11: e0147162. doi:10.1371/journal.pone.0147162

2. Endo Y, Heine SJ, Lehman DR. Culture and positive illusions in close relationships: How my relationships are better than yours. Personality and Social Psychology Bulletin. 2000;26: 1571–1586. doi:10.1177/01461672002612011

3. Brown JD, Kobayashi C. Self-enhancement in Japan and America. Asian Journal of Social Psychology. 2002;5: 145–168. doi:10.1111/1467-839X.00101

4. Chan YM. Self-esteem: A cross-cultural comparison of British-Chinese, White British and Hong Kong Chinese children. Educational Psychology. 2000;20: 59–74. doi:10.1080/014434100110380

5. Spencer-Rodgers J, Peng K, Wang L, Hou Y. Dialectical self-esteem and east-west differences in psychological well-being. Personality and Social Psychology Bulletin. 2004;30: 1416–1432. doi:10.1177/0146167204264243

6. Heine SJ, Lehman DR. Culture, self-discrepancies, and self-satisfaction. Personality and Social Psychology Bulletin. 1999;25: 915–925. doi:10.1177/01461672992511001

7. Kang S-M, Shaver PR, Sue S, Min K-H, Jing H. Culture-specific patterns in the prediction of life satisfaction: Roles of emotion, relationship quality, and self-esteem. Personality and Social Psychology Bulletin. 2003;29: 1596–1608. doi:10.1177/0146167203255986

8. Lee Y-T, Seligman ME. Are Americans more optimistic than the Chinese? Personality and Social Psychology Bulletin. 1997;23: 32–40. doi:10.1177/0146167297231004

9. Dinesen PT, Hooghe M. When in Rome, do as the Romans do: The acculturation of generalized trust among immigrants in Western Europe. International Migration Review. 2010;44: 697–727. doi:10.1111/j.1747-7379.2010.00822.x
