# Supplementary material for: Migration, acculturation, and the maintenance of between-group cultural variation
Source: PLoS One. 2018 Oct 16;13(10):e0205573. doi: 10.1371/journal.pone.0205573 (PMC6191118; doi:10.1371/journal.pone.0205573)
Supplement: S1 Fig — (PDF) [file pone.0205573.s002.pdf]

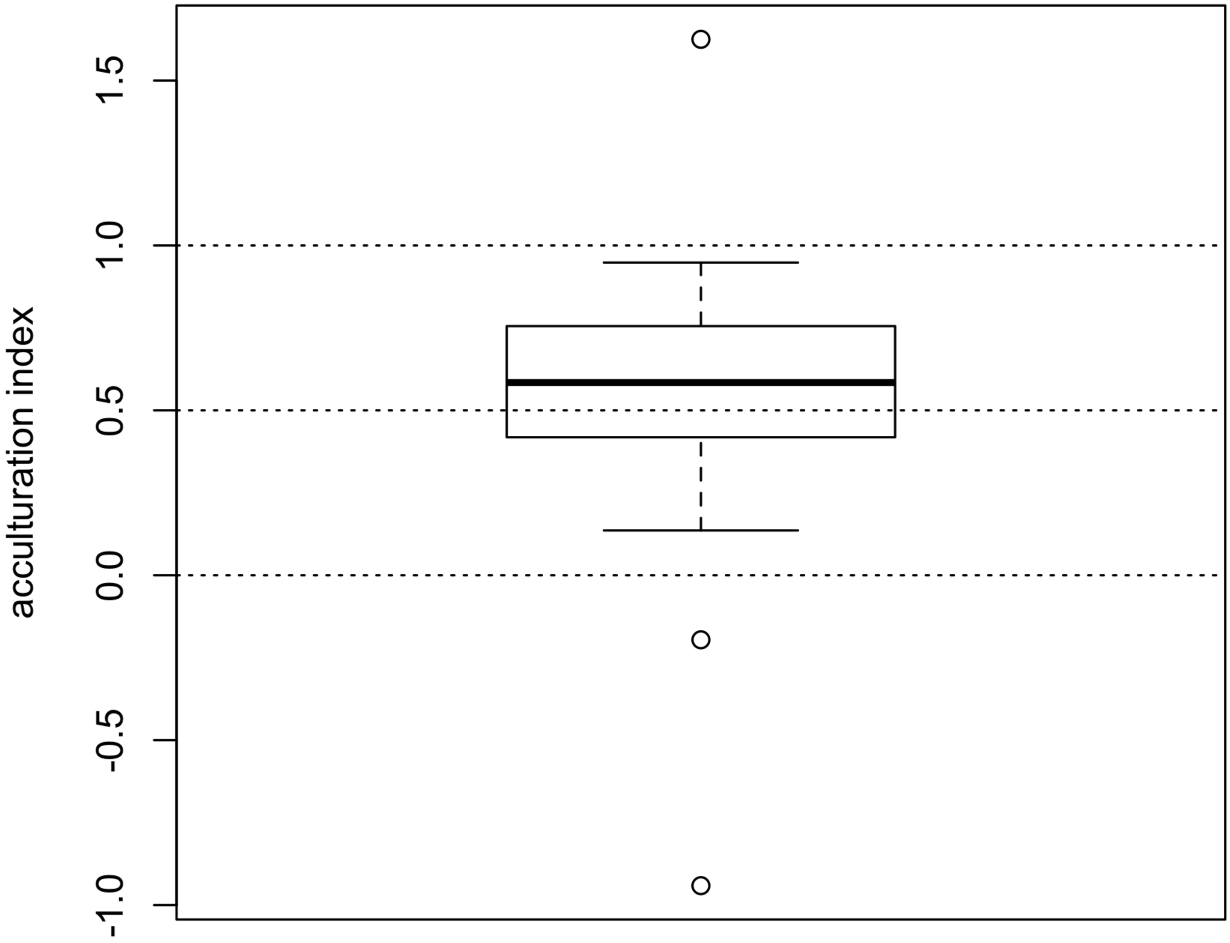

S1 Fig: Boxplot of acculturation rates. The values in S1 Table can be used to create a crude acculturation index, representing the extent of the shift in 2nd generation migrants from their 1st generation parents or heritage countries, to their host country. This is calculated as (2nd gen - host) / (1st gen or heritage - host). A value of zero indicates no acculturation, and the 2nd generation is identical to the 1st generation or heritage population. A value of one indicates complete acculturation, and the 2nd generation are identical to the host population. Note that values can fall outside 0-1 if the 2nd generation are more extreme than the 1st generation / heritage or host populations. Dotted lines show 0 (no acculturation), 1 (complete acculturation) and 0.5 (a shift 50% towards host values). This index has mean 0.54 and median 0.58, indicating a shift in 2nd generation migrants just over half of the way from their parental generation to the host country. This measure is crude given the non-systematic selection of studies for inclusion in S1 Table, and the lack of incorporation of measures of uncertainty and potentially different underlying disrtibutions for each Table S1 value used to create the index.
