# Supplementary material for: Migration, acculturation, and the maintenance of between-group cultural variation
Source: PLoS One. 2018 Oct 16;13(10):e0205573. doi: 10.1371/journal.pone.0205573 (PMC6191118; doi:10.1371/journal.pone.0205573)
Supplement: S1 Methods — (PDF) [file pone.0205573.s003.pdf]

Supporting Information for 'Migration, acculturation, and the maintenance of between-group cultural variation'

Alex Mesoudi *

*Human Behaviour and Cultural Evolution Group, Biosciences, University of Exeter, Penryn, Cornwall TR10 9FE, United Kingdom; a.mesoudi@exeter.ac.uk

S1 Methods

Calculation of F_{ST}

Wright's F_{ST} is the proportion of total population variation that occurs between sub-populations rather than within. In our models we have a population divided into s equally-sized sub-populations and s different traits. To calculate F_{ST} we first calculate the total variance, i.e. the probability that two randomly chosen individuals from the entire population have the same trait, ignoring sub-population structure. If $X_{i,j}$ is the frequency of trait i in sub-population j, and $\bar{X}_i$ is the mean frequency of trait i across all sub-populations, then the total variance, var_{total}, is given by

$$var_{total} = 1 - \sum_{i=1}^{i=s} \bar{X_i}^2$$

We then calculate the within-group variance for each sub-population, i.e. the probability that two randomly chosen individuals from that sub-population have the same trait. If var_j is the variance in sub-population j, given by

$$var_j = 1 - \sum_{i=1}^{i=s} X_{i,j}^2$$

then the overall within-population variance, var_{within}, is the mean of these variances:

$$var_{within} = \frac{\sum_{j=1}^{j=s} var_j}{s}$$

F_{ST} is then given by

$$F_{ST} = \frac{var_{total} - var_{within}}{var_{total}}$$

Conformity for more than two traits and more than three demonstrators

Boyd and Richerson [1] provide a model for the change in trait frequency under the assumption of conformist transmission, such that traits that are more frequent are more likely to be adopted relative to unbiased transmission. Their basic model (on p.208) assumes two traits (c and d) and three demonstrators (they call these 'models' but this is confusing, so I use 'demonstrators'). They use the binomial theorem to calculate the probability that different sets of three demonstrators will meet at random and pass on their traits. A parameter D, equivalent to a in the current models so henceforth labelled a, specifies the increased probability of adopting the majority trait (held by 2/3 of the demonstrators) and decreased probability of adopting the minority trait (held by 1/3 of the demonstrators), when demonstrators possessed different traits. When $a = 1$ there is maximum conformity, when $a = 0$ there is no conformity and transmission is unbiased. For two traits and three demonstrators, the frequency p' of a trait after conformity is given by

$$p' = p + ap(1-p)(2p-1) \qquad (S1)$$

where p is the frequency of the trait before conformity. This however only applies when there are three randomly chosen demonstrators and two traits. The effect of increasing a can be seen in S2 Fig. Increasing a from 0.5 to 1 increases the speed with which the initially more common trait goes to fixation.

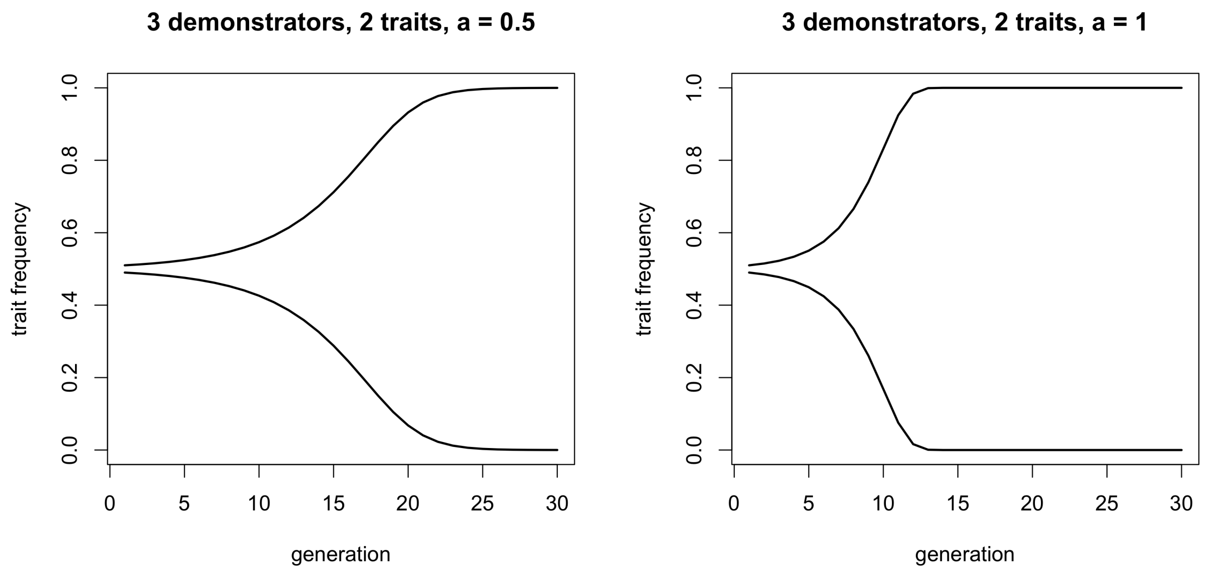

S2 Fig: The effect of conformity on trait frequencies, for two traits and three demonstrators. The trait that is initially more common (initial frequency = 0.51) than the other trait (initial frequency = 0.49) goes to fixation. This occurs faster when the strength of conformity, a, is larger.

Boyd and Richerson [1] extended their model to include more than three demonstrators, but their formulation (p.213) failed to specify a conformity function. Efferson et al. [2] provided a clearer model of conformity with more than three demonstrators, also using the binomial theorem, showing that the effect of conformity increases with the number of demonstrators. However they did not extend to more than two traits. Nakahashi et al. [3] devised a model extending conformity to more than two traits, but used a different formulation to the binomial model. Their model used a parameter also labelled a: when their $a = 1$ there is no conformity, when their $a > 1$ there is conformity, and when their $a = \infty$ there is maximum conformity. However, this model has an unclear individual level interpretation. Boyd and Richerson's [1] conformity parameter has a clear meaning: when their parameter equals 1, then individuals faced with majority and minority traits always pick the majority trait. It is unclear, however, what Nakahashi et al's [3] $a = \infty$ means in this context, particularly when one wants to generate empirically testable predictions regarding acculturation strengths and compare to an individual-based model.

Here I use multinomial theorem to extend Boyd and Richerson's [1] model to more than three demonstrators and more than two traits, and with an interpretable conformity parameter.

If p_i is the frequency of a trait in the population, then to obtain the frequency of that trait in the next generation, p'_i, after conformist transmission with n randomly forming demonstrators and s traits, we can use the multinomial theorem:

$$p'_i = \sum_{k_1,k_2,...k_s=n} \binom{n}{k_1, k_2, ...k_s} \prod_{j=1}^{s} (p_j^{k_j}) \cdot X_i \qquad (S2)$$

where $k_1, k_2...k_s$ represent all combinations of s non-negative integers such that the sum of all k values is n. For example, when $s = 2$ and $n = 3$, then there are two k values, k_1 and k_2, and four combinations of k_1 and k_2 that sum to $n = 3$: $k_1 = 3$ and $k_2 = 0$; $k_1 = 2$ and $k_2 = 1$; $k_1 = 1$ and $k_2 = 2$; and $k_1 = 0$ and $k_2 = 3$. At the moment we assume that demonstrator formation is random. X_i specifies the probability of adoption of trait i for that set of k values, and incorporates the conformity parameter a. If k_{max} is the maximum k in a combination, and π is the number of traits that have $k = k_{max}$ (so when $\pi = 1$ there is a single most-common trait, and when $\pi > 1$ there are more than one joint-most-common traits), then:

$$X_i = \begin{cases} k_i/n + a(1/\pi - k_i/n) & \text{if } k_i = k_{max} \\ k_i/n - ak_i/n & \text{if } k_i \neq k_{max} \end{cases} \qquad (S3)$$

To see how these equations work, S2 Table shows how to calculate the frequency p'_1 of trait $i = 1$ following conformity given two traits, 1 and 2 ($s = 2$) and three demonstrators ($n = 3$). The first three columns 'Dem 1-3' contain all possible combinations of traits 1 and 2 among the three demonstrators. k_1 and k_2 are the number of copies of trait 1 and 2 respectively in that row's demonstrators. 'Coef' contains the number of each combination of demonstrator traits ignoring order, and is the multinomial coefficient. The column $P(forming)$ gives the probability of that combination of demonstrators forming. This is given by the standard multinomial expression, i.e. the product of each trait frequency (p and $1 - p$, given that there are only two traits) raised to the powers k_1 and k_2 respectively.

S2 Table: Example frequency table for calculating the frequency p_1' of trait 1 following conformity given two traits, 1 and 2 ($s = 2$) and three demonstrators ($n = 3$) See text for details. Dem=demonstrator, Coef=Coefficient, P(forming)=probability of that demonstrator combination forming randomly, P(adopt 1)=probability of adopting trait 1

Dem 1	Dem 2	Dem 3	k_1	k_2	Coef	$P(forming)$	$P(adopt\ 1) = X_1$	$Coef * P(forming) * P(adopt\ 1)$
1	1	1	3	0	1	p^3	1	p^3
1	1	2						
1	2	1	2	1	3	$p^2(1-p)$	$2/3 + a/3$	$3p^2(1-p)(2/3 + a/3)$
2	1	1						
1	2	2						
2	1	2	1	2	3	$p(1-p)^2$	$1/3 - a/3$	$3p(1-p)^2(1/3 - a/3)$
2	2	1						
2	2	2	0	3	1	$(1-p)^3$	0	0

$P(adopt\ 1)$ gives the probability of that row's demonstrator trait combination resulting in the observer adopting trait 1, incorporating the conformity parameter a. This is X_i in Equation S2 and is given in Equation S3. In the first row/combination, $k_1 = 3 = k_{max} = n$ and $\pi = 1$, so $X_i = k_1/n + a(1/\pi - k_i/n) = 1 + 0 = 1$. For the next three demonstrator combinations, $k_1 = 2 = k_{max}$ and $\pi = 1$, so $X_i = k_i/n + a(1/\pi - k_i/n) = 2/3 + a(1 - 2/3) = 2/3 + a/3$. And so on for the other demonstrator combinations. The final column contains the product of the coefficient, $P(forming)$ and $P(adopt\ 1)$. The sum of this final column gives the frequency of trait 1 in the next generation, p_1':

$$p_i' = p^3 + 3p^2(1-p)(2/3 + a/3) + 3p(1-p)^2(1/3 - a/3) \quad (S4)$$

which reduces to Equation S1 because $s = 2$ and $n = 3$, as in Boyd and Richerson's [1] original formulation. When $s > 2$ or $n > 3$ the resulting recursion will not reduce to Equation S1, but is derived in the same way from Equation S2. In the case of $s = 2$, then the updated frequency of the other trait will equal $1 - p'$; when $s > 2$ then $s - 1$ traits need to be calculated using Equation S2.

Note that some combinations of $n > 3$ demonstrators will have more than one maximum k, e.g. for the trait combination {1,1,2,2,3} then $k_1 = 2$, $k_2 = 2$ and $k_3 = 1$, so k_1 and k_2 are both k_{max}. In such cases Equation S3 also applies, to increase the frequency of both most-common traits equally by the amount that the minority traits are decreased.

S3 Fig shows the effect of increasing the number of demonstrators ($n > 3$) and adding more than two traits ($s > 2$) to the change in trait frequencies over time, with no assortation ($r = 0$). Increasing n increases the speed with which majority traits go to fixation. Increasing s causes the initially most-common trait to go to fixation, and all other traits to be eliminated.

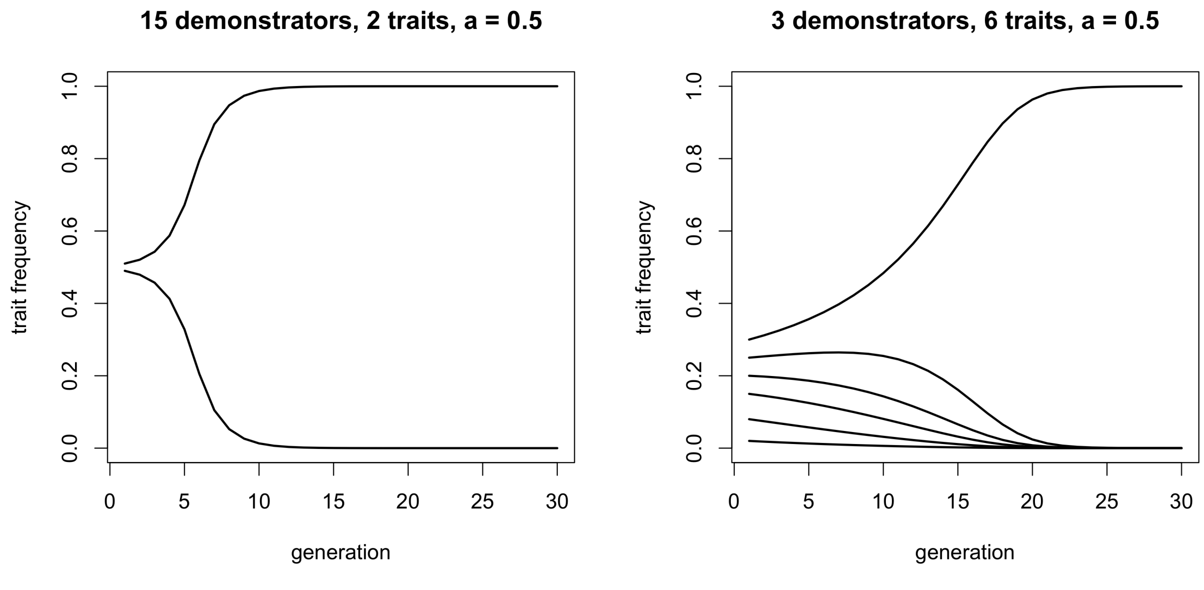

S3 Fig: The effect of conformity on trait frequencies, for more than three demonstrators (left) and more than two traits (right). Increasing the number of demonstrators increases the strength of conformity (compare with S2 Fig, left panel). For more

than two traits, whichever trait is initially most frequent goes to fixation, even if this frequency is initially less than 0.5 (here initial trait frequencies were 0.3, 0.25, 0.2, 0.15, 0.08, and 0.02). Other parameters: r=0

S4 Fig shows the probability of adopting a trait given different frequencies of that trait in the population, which have become defining images of conformity in the cultural evolution literature. I show only the case of $s = 2$ for ease of interpretation. The left panel shows that a non-zero conformity parameter a generates S-curves, such that when the trait is common (its frequency is greater than 0.5) then the probability of adoption is exaggerated, and when the trait is uncommon (less than 0.5) then the probability of adoption is decreased, relative to the dotted line which shows unbiased, non-conformist transmission. Larger values of a generate stronger conformity curves. The right panel shows that increasing n also increases the strength of conformity, keeping a constant.

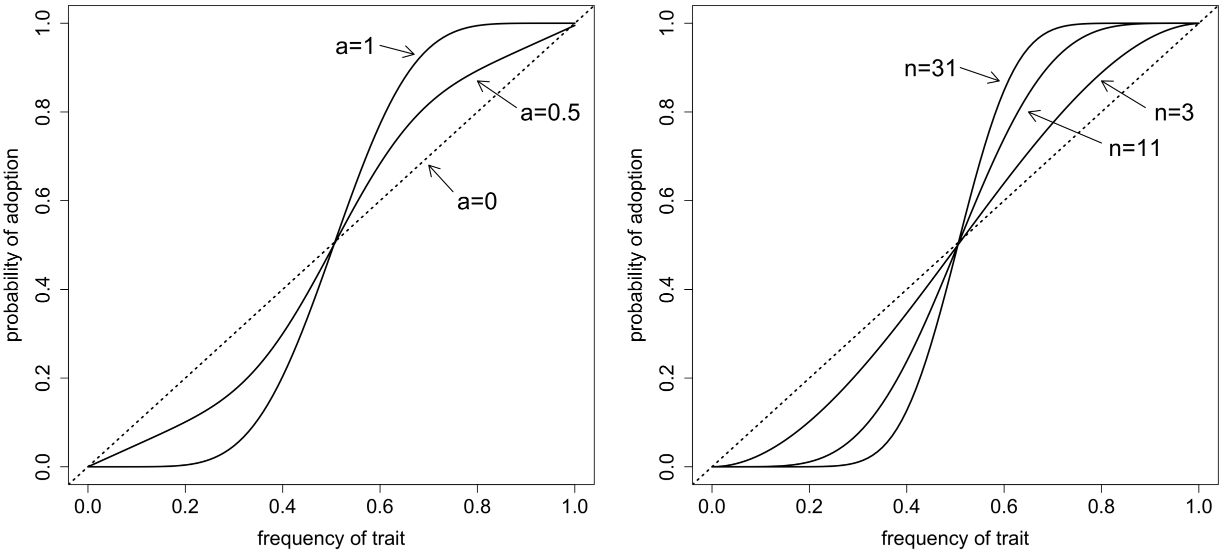

S4 Fig: How conformity affects trait adoption. The y-axis shows the probability of adopting a trait as a function of that trait's frequency in the population. Here we assume only two traits (s=2). The dotted line shows unbiased, non-conformist transmission: the probability of adoption is exactly equal to the frequency in the population. The left panel shows different values of a for constant n (n=15). The right panel shows different values of n for constant a (a=1). Other parameters: r=0.

We now add non-random formation of demonstrators, or assortation. Boyd and Richerson [1] also implemented assortation, but they restricted its effect to a correlation r between two of three demonstrators. Here I wish to add a general assortation effect across all n demonstrators, where $n \geq 3$. The simplest case would be where a fraction $1 - r$ of demonstrator combinations form randomly as specified in Equation S2, and a fraction r form culturally homogenous sets of demonstrators who all possess the same cultural trait. In S2 Table, this would be the first row (all demonstrators have trait 1) and last row (all demonstrators have trait 2). If we are interested in how p_i changes, then only one of these combinations will result in a change in p_i (the one where $k_i = n$) due to the X_i term. Assuming learners must possess the same trait p_i as the homogenous set of demonstrators, then a fraction p_i of individuals will learn from homogenous sets. Homogeneous sets always produce the same trait (again due to the X_i term), and so result in the same frequency of p_i as before transmission. In Equation S5, this is reflected in the rp_i term:

$$p_i' = (1-r)\left[\sum_{k_1,k_2,\ldots k_s=n} \binom{n}{k_1, k_2, \ldots k_s} \prod_{j=1}^{s} (p_j^{k_j}) \cdot X_i\right] + rp_i \qquad (S5)$$

It is clear from Equation S5 that as r increases, the effect of conformity via X_i becomes weaker.

Model 2 fitness plots and assumptions

The fitness functions given in the main text are a specific cooperation version of the general fitness functions for coordination games provided in Boyd & Richerson [4]. S4 Fig plots the fitness of cooperators (w_c, blue line) and defectors (w_d, red line) at different frequencies of cooperators, p, equivalent to Fig 1 in [4]. The lines cross at p^*, as given by Equation 4 in the main text. This point p^*, where the fitness of cooperators and defectors is equal, is an unstable equilibrium for payoff-biased within-group social learning (as determined by L). To ensure that fitnesses are always positive, I assume that $b > c$, $b > v$ and $c + u < 1$.

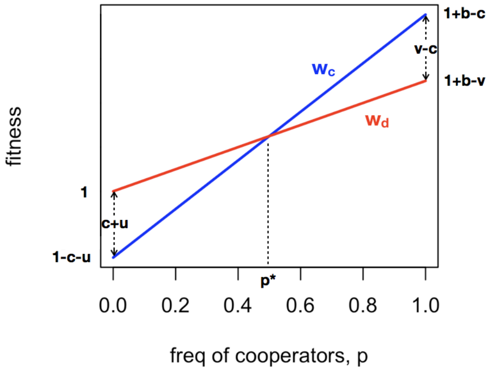

S5 Fig: Fitnesses of cooperators (w_c, blue line) and defectors (w_d, red line) at different frequencies of cooperators, p. Annotations show how fitness parameters affect these functions, and p^* indicates where the lines cross.

The fitness parameter values were chosen in S5 Fig so that $p^* = 0.5$ and the basin of attraction within which cooperators have higher fitness than defectors ($w_c > w_d$ or $p > p^*$) and the basin of attraction within which defectors have higher fitness than cooperators ($w_d > w_c$ or $p < p^*$) are equal. Other fitness parameter values give different values of p^* and alter the relative sizes of these basins of attraction. The easiest way of doing this is varying v, the punishment cost borne by defectors. S6 Fig shows fitness plots for three different values of v, in the centre with equal basins of attraction (as in S5 Fig), on the right where cooperators have a larger basin of attraction, and on the left where defectors have a larger basin of attraction. This has consequences for the action of payoff-biased within-group social learning (L) by changing the unstable equilibrium point.

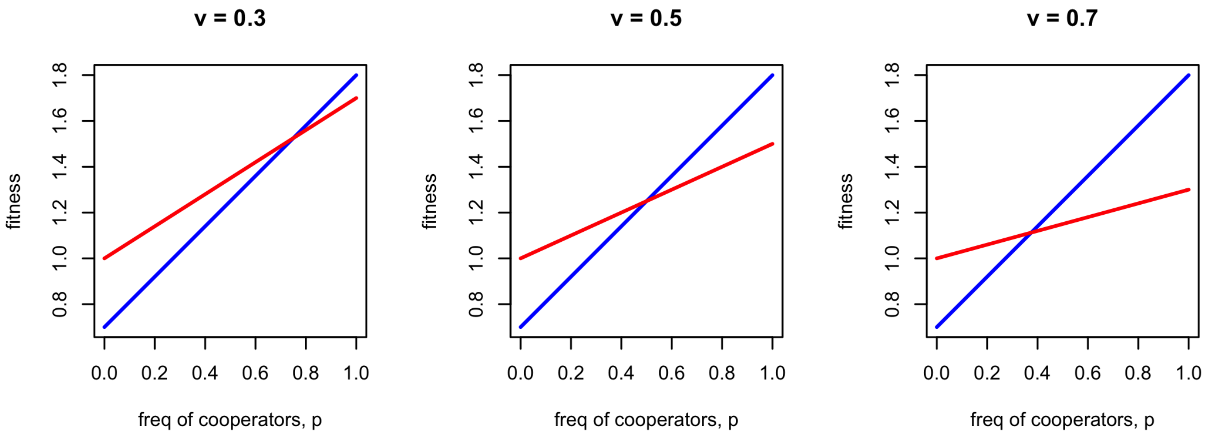

S6 Fig: Fitnesses plots for three different values of v, which determines the cost of being punished for defectors. When v is large, cooperators have a larger basin of attraction. When v is small, defectors have a larger basin of attraction. Other parameters: b=1, c=0.2, u=0.1.

Equation S6 gives the mean population fitness W. S7 Fig plots this quadratic function of p.

$$W = pw_c + (1-p)w_d = (u+v)p^2 + (b-v-c-u)p \qquad (S6)$$

To keep the model simple I assume that W is always greater than 1, in other words, a population entirely composed of defectors ($p = 0$) always has lower fitness than any population containing any cooperators ($p > 0$). In terms of S7 Fig, this would mean that the green W line never drops below $W = 1$. To ensure this, I assume that $(b-c) > (u+v)$. Finally, the parameter μ in Equation 5 ensures that the weighted migration rate $m(W-1)$ never exceeds the baseline migration rate m. This is done by setting μ to be the reciprocal of the maximum value of $W-1$ which occurs at $p = 1$, so $\mu = 1/(b-c)$. Similarly, γ in Equation 6 ensures that the change in p due to payoff-biased social learning always scales from 0 to L. From S5 Fig, the maximum absolute fitness difference between cooperators and defectors ($w_c - w_d$) is either $v - c$ or $c + u$, whichever is larger, so $\gamma = 1/[max(v-c, c+u)]$.

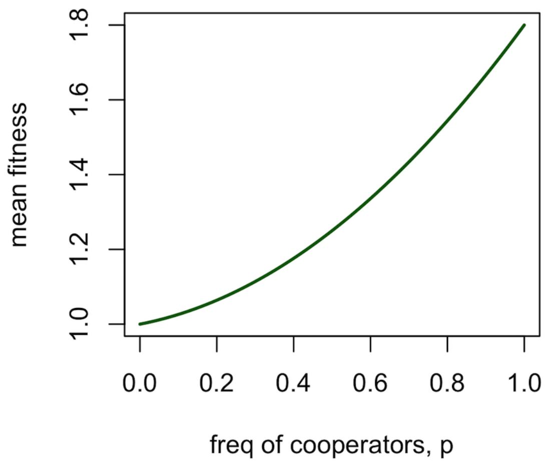

S7 Fig: Mean sub-population fitness W as a function of p, as given by Equation S5. Parameters: b=1, c=0.2, u=0.1, v=0.5.

References

1. Boyd R, Richerson PJ. Culture and the evolutionary process. Chicago, IL: Univ. Chicago Press; 1985.

2. Efferson C, Lalive R, Richerson PJ, McElreath R, Lubell M. Conformists and mavericks: The empirics of frequency-dependent cultural transmission. Evolution and Human Behavior. 2008;29: 56–64. doi:10.1016/j.evolhumbehav.2007.08.003

3. Nakahashi W, Wakano JY, Henrich J. Adaptive social learning strategies in temporally and spatially varying environments. Hum Nat. 2012;23: 386–418. doi:10.1007/s12110-012-9151-y

4. Boyd R, Richerson PJ. Voting with your feet: Payoff biased migration and the evolution of group beneficial behavior. Journal of Theoretical Biology. 2009;257: 331–339. doi:10.1016/j.jtbi.2008.12.007
