# Supplementary material for: Migration, acculturation, and the maintenance of between-group cultural variation
Source: PLoS One. 2018 Oct 16;13(10):e0205573. doi: 10.1371/journal.pone.0205573 (PMC6191118; doi:10.1371/journal.pone.0205573)
Supplement: S1 Results — (PDF) [file pone.0205573.s004.pdf]

# Supporting Information for 'Migration, acculturation, and the maintenance of between-group cultural variation'

*Alex Mesoudi* *

*Human Behaviour and Cultural Evolution Group, Biosciences, University of Exeter, Penryn, Cornwall TR10 9FE, United Kingdom; a.mesoudi@exeter.ac.uk

# S1 Results

## Individual-based version of Model 1

In order to verify the recursion-based models that track trait frequencies, I created analogous individual-based models in which individuals and their traits are explicitly simulated. These individual-based models have the same assumption of $s$ sub-populations and $s$ traits. Now, however, we specify the number of individuals in each sub-population, $N$. This is different to $n$, which is the number of demonstrators sampled during conformist acculturation. $n$ is also implemented in the individual-based models, so individuals pick $n$ demonstrators from the $N$ members of their sub-population (so $n \leq N$). The individual-based simulations start with the same complete between-group structure as the recursion models, i.e. all $N$ individuals in sub-population 1 have trait 1, all $N$ individuals in sub-population 2 have trait 2, etc. $F_{ST}$ is calculated in the same way as for the recursion-based model after calculating overall trait frequencies from individuals' traits. Migration occurs in the same way as the island model. Every time-step, each individual moves to a common migrant pool with probability $m$, and then randomly disperses across the newly vacant spots ignoring sub-population structure. Conformist acculturation is implemented slightly differently. Following all migration, each individual chooses $n$ demonstrators from within their sub-population, and with probability $a$ adopts the most common trait among those $n$ demonstrators. Otherwise they retain their existing trait. This is conceptually the same as the recursion-based conformity described above, but without the multinomial theorem implementation, thus providing a conceptual replication of conformity as implemented in the main text. For each of the $n$ chosen demonstrators, with probability $1 - r$ that demonstrator is chosen randomly from the focal individual's sub-population. With probability $r$ that demonstrator has the same trait as the focal individual. Consequently, when assortation parameter $r = 1$, then individuals only ever learn from demonstrators with the same trait as themselves. Parameter definitions for the individual-based model are given in S3 Table. Full code of all models is available in https://github.com/amesoudi/migrationmodels.

**S3 Table: Parameter definitions for Model 1 individual-based simulations**

| Parameter | Definition |
|---|---|
| $s$ | The number of sub-populations, and also the number of alternative cultural trait values |
| $m$ | Migration rate: the probability in one time-step that an individual moves to a randomly chosen sub-population (equivalently, the proportion of the population that moves in one time-step) |
| $n$ | The number of demonstrators from whom individuals learn during acculturation |
| $a$ | Acculturation rate: the probability that an individual adopts the most-common trait among $n$ demonstrators chosen from their sub-population, as opposed to retaining their existing trait. When $a = 1$, there is 100% chance of copying the most-common trait (if there is one). |
| $N$ | The number of individuals in each sub-population (giving $Ns$ individuals in the entire population) |
| $r$ | The probability that a demonstrator is chosen who has the same trait as the focal individual, rather than chosen at random. |

S8, S9 and S10 Figs show the individual-based model results equivalent to the recursion-based model results shown in Figs 1, 2 and 3 respectively. There is very little difference between the two models, despite their different implementation, increasing our confidence in the robustness of the conclusions. There is a slight tendency for $F_{ST}$ to be lower in the individual-based model than the recursion-based model, especially when diversity is low (e.g. $a = 0.6$ in S8 Fig C). This may be due to drift which operates only in the finite populations of the individual-based models. If a trait is lost due to drift, then diversity will be reduced. S11 Fig repeats Fig 2 and S10 Fig but with larger $n$ than is computationally feasible with the recursion-based model. Here we can see that increasing $n$ above 13 does not change the dynamics, except at very low values of $a$ when migration is very high (S11 Fig C).

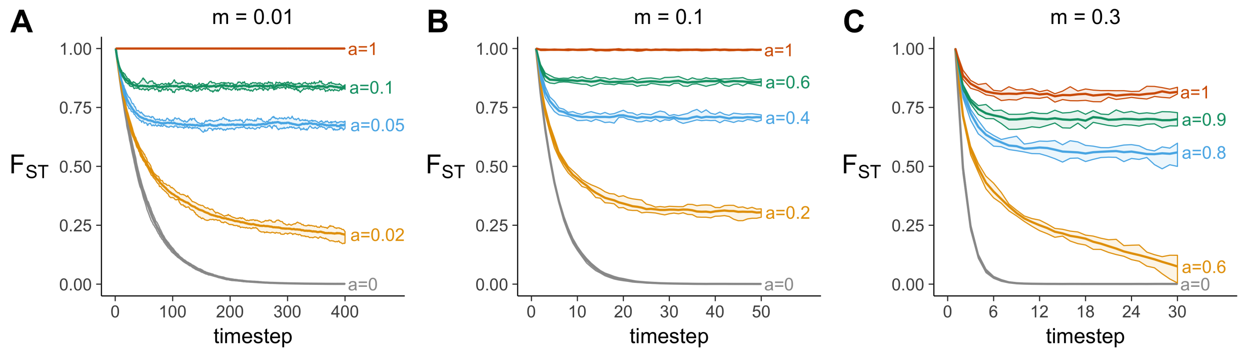


**S8 Fig: Time series showing changes in $F_{ST}$ over time for (A) a low migration rate $m$=0.01, (B) a moderate migration rate $m$=0.1, and (C) a high migration rate $m$=0.3, at varying strengths of acculturation, $a$, in the individual-based model. Other parameters: $s$=5, $n$=5, $N$=1000; results are the average of 10 independent simulation runs.**

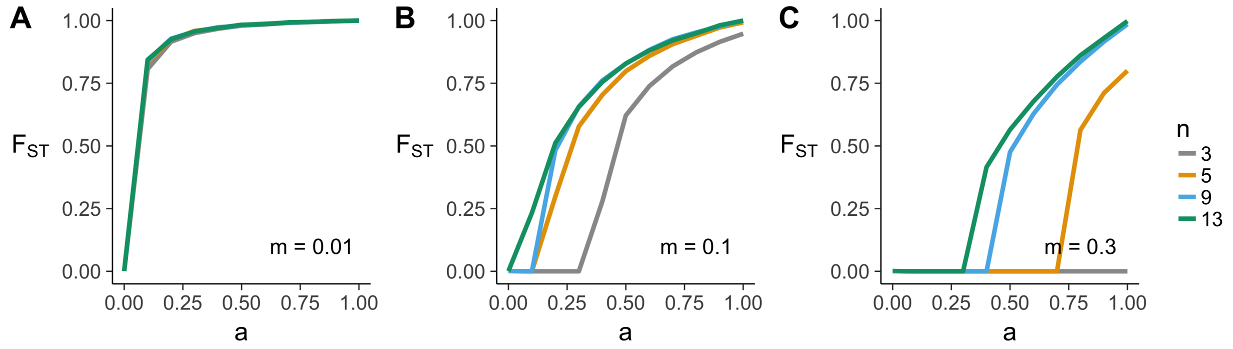


**S9 Fig: The relationship between $a$ and $F_{ST}$ at three different migration rates, and different values of $n$, for the individual-based model. Other parameters: $s$=5, 500 timesteps, $N$=1000; results are the average of 10 independent simulation runs.**

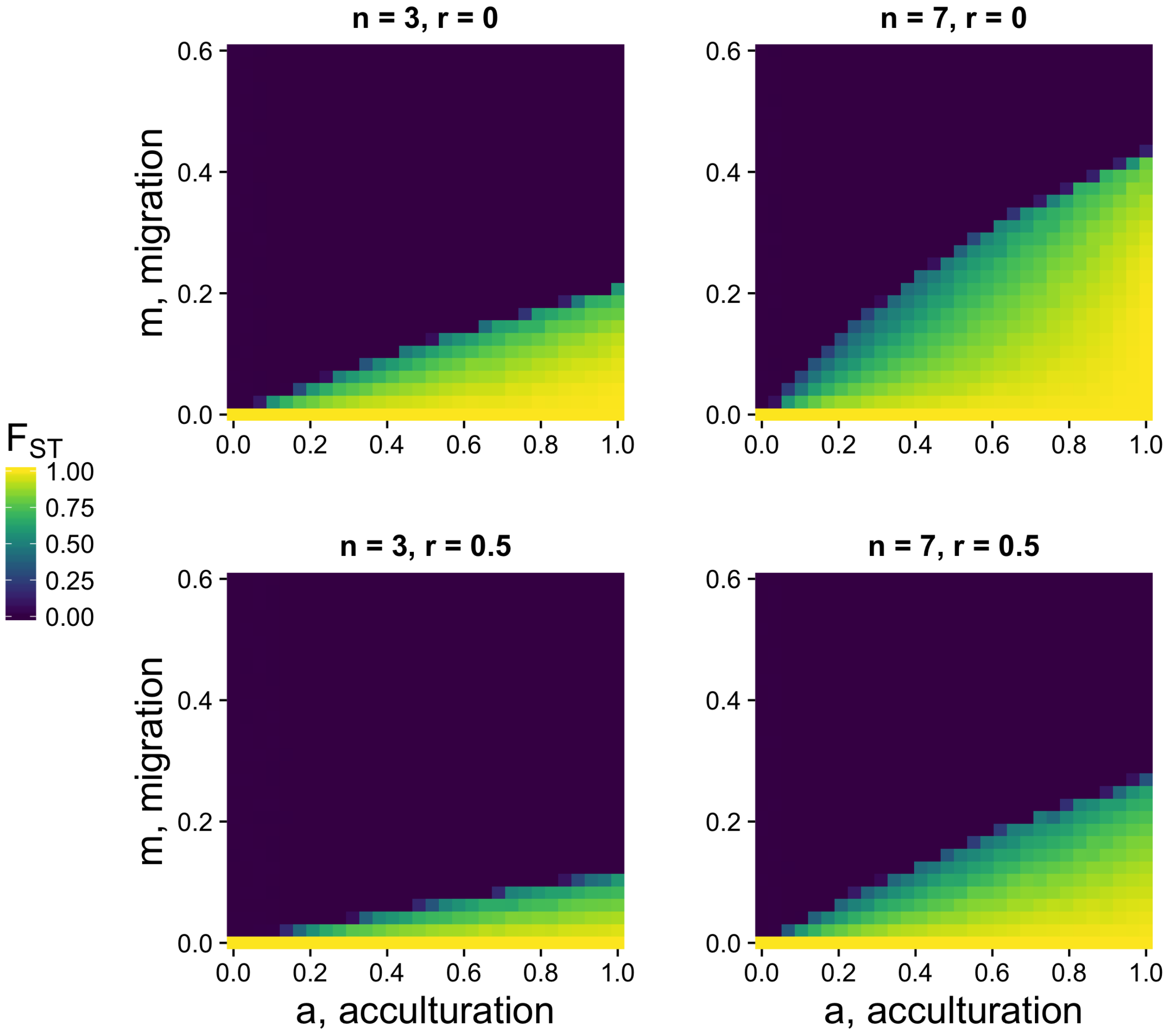


**S10 Fig: Heatmap showing $F_{ST}$ for varying acculturation rates, $a$, and migration rates, $m$, separately for three different values of $n$, the number of demonstrators, for the individual-based model. Other parameters: $s$=5, $N$=1000, 500 timesteps; results are the average of 10 independent simulation runs.**

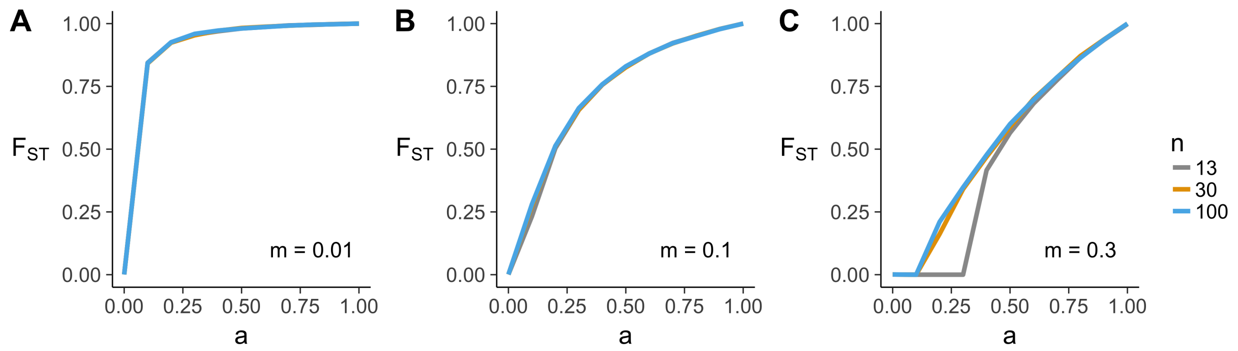


**S11 Fig: The relationship between $a$ and $F_{ST}$ at three different migration rates, for three large values of $n$, for the individual-based model. Other parameters: $s$=5, 500 timesteps, $N$=1000; results are the average of 10 independent simulation runs.**
